# Draft Genome Sequence of Sinorhizobium meliloti Strain AK170

**DOI:** 10.1128/MRA.01571-18

**Published:** 2019-01-03

**Authors:** Olga A. Baturina, Victoria S. Muntyan, Maria E. Cherkasova, Alla S. Saksaganskaya, Nikolay I. Dzuybenko, Marsel R. Kabilov, Marina L. Roumiantseva

**Affiliations:** a Institute of Chemical Biology and Fundamental Medicine, Siberian Branch of the Russian Academy of Sciences (ICBFM SB RAS), Novosibirsk, Russian Federation; b All-Russian Research Institute for Agricultural Microbiology (ARRIAM), Laboratory of Genetics and Selection of Microorganisms, Saint Petersburg, Russian Federation; c N. I. Vavilov Institute of Plant Genetic Resources (VIR), Saint Petersburg, Russian Federation; Loyola University Chicago

## Abstract

Root nodule bacteria of Sinorhizobium meliloti species live in a symbiotic relationship with alfalfa plants. We report here the draft genome sequence of S. meliloti strain AK170, recovered from nodules of Medicago orthoceras (Kar. & Kir.) growing in an area impacted by salinization.

## Abstract (precis)

Root nodule bacteria of Sinorhizobium meliloti species live in a symbiotic relationship with alfalfa plants. We report here the draft genome sequence of S. meliloti strain AK170, recovered from nodules of Medicago orthoceras (Kar.

## ANNOUNCEMENT

Sinorhizobium meliloti strain AK170 was isolated from nodules of Medicago orthoceras (Kar. & Kir.) Trautv. (syn. Trigonella orthoceras Kar. & Kir.) in the Mugodzhary region in 2002. This region is a part of a modern center of alfalfa intrоgressive hybridization in northwest Kazakhstan, which has been suffering from manmade salinization since the 1960s (1–3).

The culture of AK170 was stored in glycerol at −80°C. A glycerol stock was used to inoculate a tryptone-yeast extract (TY) agar plate (4); the resulting single colony was incubated overnight in TY broth (28°C, 180 rpm). Genomic purification was done by using a Pacific Biosciences protocol with Phase Lock Gel (VWR International) and AMPure beads (Agencourt Bioscience, Beckman Coulter Life Sciences), following the E2612 protocol from New England BioLabs.

Total DNA was fragmented with medium-sized fragments of about 600 bp in a microTUBE Adaptive Focused Acoustics (AFA) fiber snap-cap tube using a Covaris S2 instrument. The DNA library was constructed using the dual-index NEBNext multiplex oligos (NEB) and the NEBNext Ultra II DNA library prep kit for Illumina (NEB). This DNA library was sequenced with reagent kit version 3 (600-cycle) on a MiSeq platform (Illumina) at the SB RAS Genomics Core Facility (ICBFM SB RAS, Novosibirsk, Russia). The entire genome was assembled *de novo* using the SPAdes software version 3.11.1 with default parameters (5). Gap closure and scaffolding were performed using SSPACE version 3.0 (6) (-x 1 -m 15 -o 3 -n 10 -v 0 -g 0 -T 12 -S 0, and other parameters at default) and GapFiller version 1.10 (7) (-m 20 -d 100 -T 12 -i 2, and other parameters at default). Contig reordering was done using the Mauve Aligner version 2.4.0 (8). A total of 798,724 reads were assembled to a draft genome of 6,603,254 nucleotides at 28-fold coverage, with 62.2% GC content. The genome sequence consists of 82 contigs, with an *N*_50_ value of 195,324 nucleotides (nt). The NCBI Prokaryotic Genome Annotation Pipeline (9) found 6,584 protein-coding genes, 2 rRNA operons, 49 tRNA genes, and 1 transfer-messenger RNA (tmRNA).

The *oriC* sequence of AK170 (477 bp) has 100% identity with the corresponding sequences in genomes of Rm1021 (GenBank accession number AL591688) and AK83 (GenBank accession numbers NC_015590 and CP002781). The genome of AK170 does not contain any sequences similar to those which were identified as phage-related genomic islands in Rm1021 (10). Two of the three known open reading frames (ORFs) of *hsd* genes encoding a type I restriction-modification system and are known as a part of genomic island Sme19T of Rm1021 (10) were detected in genome of strain AK170. Both *hsdR* and *hsdM* of AK170 showed less than 70% nucleotide similarity with those in Rm1021 and more than 81% with the chromosome sequences of Rhizobium sp. strain TAL182 and the chromosome 2 sequence of Rhizobium sp. strain Y9 (GenBank accession numbers CP021024 and CP018000), respectively.

### Data availability.

The genome sequence of Sinorhizobium meliloti AK170 is deposited in GenBank under the accession number RDQX00000000. Raw sequencing data are registered in the NCBI SRA database under the accession number SRS3952362. This announcement describes the first version of the genome assembly.
